# Rainfall seasonality and drought performance shape the distribution of tropical tree species in Ghana

**DOI:** 10.1002/ece3.4384

**Published:** 2018-07-30

**Authors:** Lucy Amissah, Godefridus M. J. Mohren, Boateng Kyereh, Victor K. Agyeman, Lourens Poorter

**Affiliations:** ^1^ Council for Scientific and Industrial Research‐Forestry Research Institute of Ghana Kumasi Ghana; ^2^ Forest Ecology and Forest Management Group Wageningen University & Research Wageningen The Netherlands; ^3^ College of Agriculture and Natural Resources Kwame Nkrumah University of Science and Technology Kumasi Ghana

**Keywords:** drought, dry forest, physiology, species distribution, tropical forest, wet forest

## Abstract

Tree species distribution in lowland tropical forests is strongly associated with rainfall amount and distribution. Not only plant water availability, but also irradiance, soil fertility, and pest pressure covary along rainfall gradients. To assess the role of water availability in shaping species distribution, we carried out a reciprocal transplanting experiment in gaps in a dry and a wet forest site in Ghana, using 2,670 seedlings of 23 tree species belonging to three contrasting rainfall distributions groups (dry species, ubiquitous species, and wet species). We evaluated seasonal patterns in climatic conditions, seedling physiology and performance (survival and growth) over a 2‐year period and related seedling performance to species distribution along Ghana's rainfall gradient. The dry forest site had, compared to the wet forest, higher irradiance, and soil nutrient availability and experienced stronger atmospheric drought (2.0 vs. 0.6 kPa vapor pressure deficit) and reduced soil water potential (−5.0 vs. −0.6 MPa soil water potential) during the dry season. In both forests, dry species showed significantly higher stomatal conductance and lower leaf water potential, than wet species, and in the dry forest, dry species also realized higher drought survival and growth rate than wet species. Dry species are therefore more drought tolerant, and unlike the wet forest species, they achieve a home advantage. Species drought performance in the dry forest relative to the wet forest significantly predicted species position on the rainfall gradient in Ghana, indicating that the ability to grow and survive better in dry forests and during dry seasons may allow species to occur in low rainfall areas. Drought is therefore an important environmental filter that influences forest composition and dynamics. Currently, many tropical forests experience increase in frequency and intensity of droughts, and our results suggest that this may lead to reduction in tree productivity and shifts in species distribution.

## 1 INTRODUCTION

Tropical forests are under threat of longer and intense drought as a result of global climate change. This trend is expected to continue as “many IPCC‐AR4 models predict reduced precipitation and long‐term soil moisture droughts in some tropical areas” (Fauset et al., 2012; Sheffield & Wood, 2008). However, the specific impact of drought on the distribution of tree species in many tropical forests is largely unknown. Understanding factors that shape species distribution will provide baseline information for predicting the potential impact of climate change on tropical forests. Across the globe, water availability is one of the main factors shaping the distribution and diversity of plant species (Esquivel‐Muelbert et al., 2016; Fayolle et al., 2014). Seedlings are especially vulnerable to drought, because with their small root system they have limited access to soil water. Seedling drought performance is therefore thought to explain the distribution of tree species. Two alternative hypotheses may explain species distribution along water availability gradients: (a) dry forest species, hereafter referred to as “dry species” may have a better growth and survival performance in dry conditions, and wet forest species, hereafter referred to as “wet species” may have a better performance in wet conditions (Baltzer & Davies, 2012; Baltzer, Davies, Bunyavejchewin, & Noor, 2008), or (b) there may be a trade‐off between drought survival and growth performance (the growth‐productivity trade‐off, McGill, Enquist, Weiher, & Westoby, 2006), in which species ability to tolerate and survive dry conditions comes at the expense of a reduced growth in wet conditions. Hence, dry species may outperform wet species in dry environments because of higher drought survival, and wet species may exclude dry species from wet environments because of faster growth (Baltzer et al., 2008).

Species responses to drought are, among others, influenced by gas exchange and plant hydric status (i.e., their water potential), as they determine carbon gain and growth, and the ability to survive drought. Plants employ two strategies (isohydric and anisohydric) in response to water stress, which influence their drought tolerance (Attia, Domec, Oren, Way, & Moshelion, 2015; Roman et al., 2015). Isohydric species maintain tight control over their stomata during drought and close their stomata at relatively high (less negative) leaf water potential to avoid further water loss and maintain their hydric status. Such early stomatal closure comes at the expense of a reduced carbon gain and dry season growth (Sade, Gebremedhin, & Moshelion, 2012). Anisohydric species, on the other hand, keep their stomata open during drought, leading to reduced leaf water potential. These species should therefore be able to tolerate low leaf water potential (more negative) (Sade et al., 2012). An anisohydric strategy may be beneficial under moderate water stress, but may potentially lead to wilting, desiccation, and reduced survival under severe water stress (Sade et al., 2012).

Species distribution along rainfall gradients may not only be shaped by drought, but also by other environmental factors, as an increase in rainfall is associated with an increase in pest pressure and a decrease in soil fertility and irradiance (Brenes‐Arguedas, Coley, & Kursar, 2009; Coley & Barone, 1996; Gaviria & Engelbrecht, 2015; Gaviria, Turner, & Engelbrecht, 2017; Swaine, 1996). To get insight into how drought responses shape species performance and distribution along rainfall gradients, a reciprocal planting experiment is needed, in which dry and wet species are planted in dry and wet forest sites, and species performance is monitored over the dry and wet seasons. Yet, to our knowledge only five reciprocal transplanting experiments that evaluate drought performance and species distribution have been carried out in tropical forests (Baltzer & Davies, 2012; Brenes‐Arguedas et al., 2009; Gaviria & Engelbrecht, 2015; Gaviria et al., 2017; Swaine et al., 1997), which makes it difficult to generalize results. Moreover, most of these studies have been carried out at the higher end of the rainfall gradient (1,800–3,000 mm/year in Panama, 1,950–2,700 mm/year in Malaysia), where drought adaptations may be less important depending on the strength of dry seasons. In contrast, in Africa, most forests experience considerably less rainfall than other locations in the tropics. This together with the extinction of many wet forest species during the ice ages (Richards, 1973) may have led to a stronger drought‐tolerant flora in Africa compared to other continents (Holmgren & Poorter, 2007; Parmentier et al., 2007).

Here, we present results of a reciprocal transplanting experiment in Ghana, where seedlings of 23 species with contrasting distributions along a rainfall gradient (dry, ubiquitous, and wet) were planted in gaps in a dry and a wet forest site. Seasonal variation in environmental conditions (rainfall, soil water potential, irradiance, vapor pressure deficit) and seedling physiology, survival, and growth were monitored for a 2‐year period. We addressed the following questions and corresponding hypotheses:

1. How do environmental conditions vary between forest sites in the dry and wet seasons? We tested the premise that because the dry forest experiences lower rainfall, and less cloud and canopy cover and a higher irradiance compared to the wet forest (cf. Wright & Van Schaik, 1994) there will be a stronger soil water stress and atmospheric drought in the dry forest, and this difference will be more pronounced in the dry season (cf. Costa et al., 2010).
2. How do seedling physiology and performance differ between forest sites? We hypothesized that, because of increased drought stress, seedlings growing in the dry forest site will have lower stomatal conductance, leaf water potential, growth, and survival than seedlings growing in the wet forest site.
3. How do species with different distribution types differ in their physiological response and performance? We hypothesized that species will have a home advantage and that in the dry forest the dry species can cope with increased drought by having an anisohydric strategy; that is, they will keep their stomata open and have higher stomatal conductance in comparison with the wet species, leading to a more negative leaf water potential (cf. de Sade et al., 2012). As a result, they will have higher growth and survival rate than the wet‐distribution species. In wet forest, the reverse will be the case, with the ubiquitous species being in between.
4. Can relative drought performance in the field explain a continuum of species distribution along the rainfall gradient in Ghana? We hypothesized that species that exhibit higher drought survival and growth in the dry forest relative to the wet forest will occur at the drier part of the rainfall gradient (cf. Engelbrecht et al., 2007).

## 2 MATERIALS AND METHODS

### 2.1 Study sites

The study was conducted in the tropical lowland forests of Ghana. Ghana's forest vegetation is classified into seven main forest types, namely wet evergreen, moist evergreen, upland evergreen, moist semideciduous, dry semideciduous, southern marginal, and southeast outliers forests (Hall & Swaine, 1976). The forests are characterized by a rainfall gradient which varies from <750 mm to >2,000 mm/year. Within the forest zone is a network of forest reserves. According to Fauset et al. (2012) annual rainfall in some forest reserves has reduced by about 165 mm (post‐1970). The study was conducted in two forest reserves with contrasting rainfall conditions: the dry Afram Headwaters Forest Reserve (Supporting Information Appendix S1a) and the wet Neung South Forest Reserve (Supporting Information Appendix S1b). Afram Headwaters Forest Reserve is classified as a dry semideciduous forest inner zone subtype (Hall & Swaine, 1981). It covers an area of 201 km^2^ and lies on longitude 1°32′W and 1°48′W and latitude 6°45′N and 7°25′N. The altitude varies from 274 to 412 m above sea level. The reserve is characterized by uniform high temperatures and two peak rainfall seasons in June and October and a dry season from November (or December) to February. Mean monthly dry season rainfall from 1973 to 2009 was 28 mm ±13.5. Mean annual rainfall at Afram headwaters from 1973 to 2009 was 1,290 mm ±221 (Ghana Meteorological Service records). Mean maximum temperature is 30.6 ± 0.24°C and mean minimum temperature is 21.2 ± 0.23°C. Over most of the area the soil is sandy loam with patches of clay (Forestry Division, 1963).

The Neung South Forest Reserve is classified as wet evergreen forest (Hall & Swaine, 1981). The reserve covers an area of 113 km^2^ and is located between longitude 1°55′W and 2°07′W and latitude 05°06′N and 5°11′N. The reserve consists of small hills and ridges with an average altitude of about 150 m above sea level. The area is characterized by double maxima rainfall starting from March to October with two peaks in June and October and a dry season from November (or December) to February. Mean monthly dry season rainfall from 1973 to 2011 is 82.6 mm ±25.9. Mean annual rainfall from 1973 to 2011 is 1,808 mm ±293 (Ghana Meteorological Service records). Temperatures are fairly uniform and range between 26°C (August) and 30°C (March). The soil texture in most parts of the reserve is loamy clay (Forestry Commission, 2007).

### 2.2 Species selection and experimental design

Twenty‐three tree species were selected based on their contrasting distribution (see Supporting Information Appendix S2) and assigned to three rainfall distribution types based on earlier classifications (Hall & Swaine, 1981; Hawthorne, 1995, 1996; Hawthorne & Jongkind, 2006); dry forest species (10 species), a mix of moist and wet forest species (eight species), hereafter referred to as “wet” species, and ubiquitous species that occur in both dry and wet forests (five species). Most of the species selected are characteristic species of the various forest types in Ghana and achieve various frequencies in 155 25 × 25 m sample plots of Hall and Swaine (1981). For example, *Heritiera utilis* (54%) and *Pentadesma butyracea* (62%) achieve high frequency in 155 25 × 25 m sample plots in the wet evergreen forest type of Ghana. *Celtis zenkeri* (60%) *achieves* high abundance in dry semideciduous forests. *Turraeanthus africanus* (38%) *and Strombosia pustula* (100%) are characteristic species of the moist semideciduous and moist evergreen forest type, respectively. The species did not only differ in distribution type, but also in their shade tolerance. Species have previously been classified as shade tolerants (“shade bearers, sensu Hawthorne, 1995,” are understory and subcanopy species whose seeds germinate and seedling establish in forest shade), nonpioneer light‐demanding species (seeds germinate in shade but seedlings need light to establish and grow) and pioneer species (seeds germinate in gaps and seedling grow in gaps). Overall the study species comprised mostly gap‐dependent species with a higher representation of nonpioneer light‐demanding (NPLD) and pioneer species than shade‐tolerant species. The gap species were similarly distributed within the species rainfall distribution groups (Kruskal–Wallis *χ* ^2^ = 0.426, *p *=* *0.808). The dry species comprised 90% gap species versus 10% shade‐tolerant species. The wet species was composed of 75% gap species versus 25% shade‐tolerant species. In the ubiquitous group, there were 80% gap species versus 20% shade‐tolerant species.

Seeds of the 23 species (Supporting Information Appendix S2) were collected from the dry forest (Afram Headwaters Forest Reserve), moist forest (Bobiri and Pra‐Anum Forest Reserves) and wet forest (Neung South and Subri Forest Reserve) from November 2010 to March 2011, from 4 to 5 seed trees per species. For each species, the seeds from the 4 to 5 trees were mixed together before sowing. Most (70%, 7 of 10 species) of the dry forest species seeds were collected from Afram Headwaters Forest Reserve and 88% (7 out of 8 species) of the wet species were collected from wet forest (Neung South) and moist forest (Bobiri Forest Reserve). For the ubiquitous *Terminalia superba* Engl. and Diels, and *Terminalia ivorensis* A. Chev seeds were collected from Afram Headwaters Forest Reserve, for *Antiaris toxicaria* Leschenault and *Strombosia pustula* J. Leonard from Bobiri Forest Reserve. In the case of the ubiquitous *Terminalia ivorensis* A.Chev another genotype was collected from the wet forest site.

Seeds were germinated in germination trays and after 2–3 weeks seedlings were transplanted to individual polythene bags that were 12.7 cm wide and 20.3 cm long. Polybags were filled with soil collected from a moist semideciduous forest (Bobiri Forest Reserve). The seedlings grew for 2–4 months in a shade house of ca. 15% full irradiance before they were transplanted in the field. Due to unavailability of seeds during the fruiting period, seedlings of *T. africanus* (Welw. ex C.DC.) Peller, *Lophira alata*, Banks ex Gaertn., *Strombosia pustulata* J. Leonard and *Nesogordonia papaverifera* (Hook.f.) Brenan from previous seed batches were used. These seedlings of the four species grew for 10–12 months in the shade house. For these species, seedlings were grown in large poly bags of 19 cm wide and 46 cm long. The 15% irradiance enhances maximum growth of seedlings at the seedling stage (Agyeman, Swaine, & Thompson, 1999). Seedlings were watered daily in the mornings during the period they grew in the shade house.

For the transplanting experiment, fifteen naturally occurring gaps were selected for each site (Supporting Information Appendix S1a,b). Species were planted in gaps rather than the understory because in gaps both light‐demanding and shade‐tolerant species can grow and survive, besides forest dynamics is largely driven by gap‐phase regeneration (Brokaw, 1985; Feeley et al., 2007). To obtain on average 20% irradiance for each plot additional small trees were felled from the selected gaps. Irradiance measurements (in Lux) were conducted in each gap over a period of 2–4 weeks with a Fisher Scientific Traceable Dual Display light meter (Fisher Scientific Company, IL, USA). These measurements were conducted from 8 am to 4 pm daily in each of the 15 plots per forest site for a period of 2–4 weeks. In each plot, three measurements were taken daily. Concurrent measurements of irradiance were made at an open space outside the forest. Irradiance level in each plot was calculated as a percentage of irradiance of the open space outside the forest. Daily averages were calculated and the average of the 2–4 weeks period was taken as the irradiance of each plot.

Plot sizes for the planting trial were 10 × 9 m for 13 plots per site and 12 × 8 m for two plots in each site. These plot sizes were the sizes used for the planting trial but the gap border started at ca. 2 m from the trial edge. The slope of each plot was measured for all the plots using a clinometer (Clino Master‐Sisteco Precision, Finland). Average slope of plots in the dry forest was 10.1% (std. = 3.1) and in the wet forest was 12.7% (std. = 4.7). Plots were on average 72 m (std. = 47) apart in the wet forest and 74 m (std. = 34) apart in the dry forest. Plots were oriented east–west to ensure that the diurnal course of the sun had a similar effect on all gaps.

Seedlings were planted at a planting distance of 1 m in a complete randomized design with four replicates per species. The only exception was *Tieghemella heckelii* Pierre ex Chev, which had one individual per species in each plot. To minimize planting damage, seedlings were planted with portion of the soil that was in the plant bag, after removing the bag. Individual seedlings were planted in June 2011, first in the dry forest and a week later in the wet forest. A total of 2,670 seedlings were planted: (2 forests × 15 gaps × 22 species × 4 individuals per species) + (2 forests × 15 gaps × 1 species × 1 individual per species). Average seedling height of species at planting was 24.5 cm (range 8.5–63.4 cm). Plots were weeded three times in a year to remove competition from other vegetation. Survival of species was assessed 2 months after planting and dead seedlings were replaced.

### 2.3 Environmental data

Variation in seedling survival and growth between sites and seasons may be explained by variation in plant water availability, which is determined by rainfall amount and distribution, the dryness of the air (which is in turn, determined by temperature, relative air humidity, and vapor pressure deficit), soil water potential, and irradiance.

To monitor monthly rainfall a manual rainfall gauge was installed about 3 km from each forest in June 2011 and rainfall readings were taken whenever it rained. Annual rainfall in 2012 was 1,760 mm in the wet forest and 967 mm in the dry forests. In 2012, the length of the dry season (defined as months with less than 100 mm rain) was 5 months in the dry forest (Supporting Information Appendix S3a), and 2 months in the wet forest (Supporting Information Appendix S3b). In 2013, the length of the dry season was 3 months in the dry forest and 1 month in the wet forest (a short dry period in February; Supporting Information Appendix S3b). The length of dry season observed within the 2‐year study period varied from the reported dry season length of 3 or 4 months for both dry and wet forest zones in Ghana (Hall & Swaine, 1981).

In each plot, photosynthetically active radiation (PAR, the irradiance between 400 and 700 nm that is important for photosynthesis) was measured at each census, during the two dry seasons and one wet season of the experiment. PAR was measured with LI‐190SA Quantum Sensor (LI‐COR INC., Lincoln, Nebraska, USA) placed both outside (under full sunlight) and inside the plots. At each census and site, PAR was measured in all 15 plots. A quantum sensor and LI‐1400 data logger (LI‐COR INC.) were placed for 10 min at the center of each plot and the average PAR during these 10 min was logged. Measurements were conducted in the morning and in the afternoon for all 15 plots in 2–3 consecutive days. A LI‐1000 data logger (LI‐COR INC.) was used to take instantaneous PAR readings outside the plots under full sunlight. Simultaneous readings of temperature and relative humidity were made alongside PAR measurements.

To monitor relative humidity and temperature in the plots, in January 2012 (6 months after planting) in each forest, HOBO Prov2 temperature/relative humidity data loggers (Onset Computer Corporation, USA) were installed at the center in two plots (plots 1 and 10 in the wet forest, and plots 3 and 7 in the dry forest) and outside in an open area about 1 km from the forest. The resolutions were 0.02°C at 25°C for temperature, and 0.05% for relative humidity.

Simultaneous readings of temperature and relative humidity (RH) in the plots were used to calculate vapor pressure deficit (VPD) in the plots. Vapor–pressure deficit has been found to be a more accurate measure to predict plant transpiration and water loss than relative humidity. An existing look‐up table was used to ascertain the saturated vapor pressure (SVP) for a given temperature and then VPD was calculated as: VPD = ((100 − RH)/100)*SVP.

To quantify plant water availability we used soil water potential as a proxy. Soil water potential was measured using the filter paper technique (Deka et al., 1995). Soil samples were taken from 11 plots per forest for two dry seasons (January 2012 and January 2013) and a wet season (July 2012). In each plot, soil samples were taken at five positions (four corners and the center of the plot) and five depths (10, 20, 30, 40, and 60 cm), providing a total of 1,650 soil samples (2 sites × 11 plots × 5 positions × 5 depths × 3 time periods). Two batches of the Whitman 42 filter papers were used and not individually calibrated because Deka et al. (1995) found reasonable agreement between calibration curves developed for different batches of Whatman no. 42 filter papers. Plastic containers were half‐filled with collected soil samples and covered with three filter papers (Whitman 42, 55 mm, batch nos. 5365518 and 9118643) (GE Healthcare, Limited, UK) and topped with another half of the soil sample. The soil was firmly pressed to prevent air pockets from forming and hermetically sealed with black tape (PVC tape for electrical insulation, 0.13 × 19 mm, Detat Industries Corporation, China). The soil was incubated for 7–14 days. After the incubation period filter papers were carefully removed, weighed and dried at 105°C for 24 hr and reweighed. With the dry mass of the filter paper known, the moisture content of filter paper (FMC) was used to estimate soil matric potential in line with the protocol described by Deka et al. (1995) in which;

$${\text{Log}}_{10}\left(-\psi p\right)=5.144-6.699*\text{FMC},\;\text{if}\;\psi p<-51.6\;\text{kPa}$$

$${\text{Log}}_{10}\left(-\psi p\right)=2.383-1.309*\text{FMC},\;\;\text{if}\;\psi p>-51.6\;\text{kPa}$$

To quantify soil fertility, a bulk sample was made from five samples taken at depth of 20 cm (because in tropical forests most tree roots are concentrated within the first 30 cm of the soil profile) from all plots in each forest (i.e., equal amount of soil was taken from each plot sample and mixed together). Soil pH and nutrient (base saturation, cation exchange capacity, available P, K, nitrogen and carbon) analyses were carried out at the laboratory of the CSIR‐Soil Research Institute of Ghana. Soil analysis was conducted using standard laboratory procedures. Soil pH was measured in a 1:1 soil–water ratio using a glass electrode (H19017 Microprocessor) pH meter. Soil organic carbon was determined by the modified dichromate oxidation method of Walkley–Black (Nelson & Sommers, 1982). Total nitrogen was determined by the Kjeldahl digestion and distillation procedure (Bremner and Keeney 1965). Exchangeable bases (calcium, magnesium, potassium and sodium) were determined in 1.0 M ammonium acetate (NH_4_OAc) extract (Thomas, 1982). The readily acid‐soluble forms of phosphorus were extracted with HCl: NH_4_F mixture (Bray's no. 1 extract) and determined colorimetrically by ascorbic reduction (Bray & Kurtz, 1945; Olsen & Sommers, 1982) and potassium was determined by flame photometry.

### 2.4 Seedling performance and leaf physiology

We evaluated three measures of growth: height indicates the ability of the plant to compete for light, diameter is a good indicator of overall plant biomass, and leaf number indicates potentially the ability of the plant to capture light. Height, diameter and leaf number (for compound leaves, we counted the leaves and not leaflet) were measured for each seedling every 2 months throughout the 24‐month period, except between the last two measurements where the interval was 4 months. At each census survival and mortality were assessed. Seedlings were considered to be dead if the stems were brown and there was necrosis on all leaves, extensive leaf curling, and brittle leaf blades (cf. Gerhardt, 1993; Tyree, Engelbrecht, Vargas, & Kursar, 2003). At the next census seedlings that were recorded dead were rechecked; if a species had resprouted after being recorded as dead, data were corrected. Height was measured as the vertical distance between the forest floor and the apex. Stem diameter was consistently measured for each seedling at 5 cm height from ground level (for initially small seedlings) or 10 cm height (for initially taller seedlings).

We measured leaf water potential as an indicator of plant hydric status; predawn leaf water potential indicates the soil‐related water stress, and mid‐day leaf water potential the maximum soil and atmospheric drought stress the plant experiences (Ritchie & Hinckley, 1975). The leaf stomatal conductance is an indicator of gas exchange and potential carbon capture (Ewers, 2013; Veenendaal, Swaine, Agyeman et al., 1996). Leaf water potential was measured using the pressure bomb technique (Tyree & Hammel, 1972; SKPM 1405/80, Skye Instruments Ltd, UK). Stomatal conductance was measured with a leaf porometer (Model SC‐1; Decagon Devices, Inc. USA) on attached leaves. Leaf physiology (leaf water potential and stomatal conductance) was measured for the two dry seasons (January 2012 and January 2013) and one wet season of June 2012 in 11 plots per forest. These plots were randomly selected after stratification of the plots to capture the variation in habitat in the forest. The same plots were measured in the dry and wet seasons. For the wet season, stomatal conductance was measured in fewer plots (five plots per site) because cloudiness of the weather made measurements difficult (as RH was too high leading to hysteresis). For stomatal conductance measurements, two healthy‐looking individuals per species per plot were randomly sampled. For the first individual, a leaf sample was taken to determine the leaf water potential. The leaf was cut with a blade and immediately put in a transparent plastic bag. It was then placed in the pressure chamber and the pressure was adjusted until sap came out of the cut end of the petiole (Tyree & Hammel, 1972).

In total ca. 2,484 leaf conductance measurements were made (2 forests × 2 dry seasons × 11 plots × 23 species × 2 seedling per species + 2 forests × 1 wet season × 5 plots × 23 species × 2 seedlings) and 1,518 leaf water potential measurements (2 forests × 2 dry seasons × 11 plots × 23 species × 1 seedling per species + 2 forests × 1 wet season × 11 plots × 23 species × 1 seedlings).

Leaf water potential was measured at both predawn (from 5:30 to 6:30 a.m.), and around mid‐day (from 11:30 a.m. to 2:00 p.m.). In the wet season when the weather was cloudy and dark, leaf water potential measurement was extended to around 8:30 a.m. Stomatal conductance was measured throughout the day from 8:30 a.m. to 3 p.m., but for each seedling separately, at a single moment in time. There was a homogeneous distribution of measurements throughout the day for each species. When the morning was cloudy measurement started at 10:00 or 11:00 a.m. Measurements were extended to 3 p.m. to ensure the measurements in the dry and wet forests were carried out within similar time frame. Measurements were made at the abaxial surface (Willmer & Fricker, 1996) because that is where most stomata are located. The measurements of leaf water potential and stomatal conductance were made on the same individuals for the two dry seasons and the wet season.

### 2.5 Species response curves

To quantify species position along Ghana's rainfall gradient, we used the species climatic response constructed by Amissah, Mohren, Bongers, Hawthorne, and Poorter (2014). Climatic response curves were constructed for each of 18 species (there were no available data for the construction of species response curves for five species) using data from the Ghanaian national forest inventory, and four climatic variables; annual rainfall, rainfall seasonality, temperature seasonality and isothermality obtained from the WorldClim database (Hijmans, Cameron, Parra, Jones, & Jarvis, 2005). The forest inventory data consist of 2,505 1‐ha plots across forest of Ghana (Hawthorne, 1995, 1996; Hawthorne & Abu‐Juam, 1995) in which trees >5 cm stem diameter were measured in a nested design, and identified to species. For each of the species, a forward multiple logistic regression analysis of presence/absence on the four climatic variables and their quadratic terms was conducted. Parameters that were derived from the species‐specific regression models were used to calculate for each species the probability of occurrence versus annual rainfall, while keeping the other three climatic factors constant at their average value across the 2,505 1 ha plots. From these values, species rainfall response curve was constructed, and the minimum (rainfall at the 10th percentile) and optimum rainfall (rainfall value at which species occurrence reached the maximum) at which each species occurs were calculated (see Amissah et al., 2014 for details). The optimum and the minimum rainfall values calculated from the individual species response curves were taken as the species position on the rainfall gradient in Ghana.

### 2.6 Data analysis

For each seedling, absolute growth rate (AGR) in height, diameter, and leaf number were calculated as the difference in growth between the last census (at 2 years) and the first census at planting divided by the time difference. Relative growth rate (RGR) was calculated as the difference between the natural ln‐transformed values for individuals at the last census (2 years) and at planting divided by the time difference. Absolute and relative growth rates were significantly positively correlated (*r *=* *0.8, *p *<* *0.001, for height and diameter and *r *=* *0.9, *p *<* *0.001 for leaf number, Supporting Information Appendix S8), and in the main text we will focus on absolute growth rates as they better reflect species ability to compete for light. The third hypothesis was that species have a home advantage. As a result, species distribution type (dry, ubiquitous, wet–moist) was included as a fixed factor in growth and physiological variable analyses.

Repeated measures ANOVA were used to analyze the effects of forest type and season on environmental variables (PAR, temperature, relative humidity, VPD, soil matric potential) and leaf physiology (leaf water potential and stomatal conductance). Linear mixed‐effects model was used to determine the effects of forest sites and species distribution type on absolute growth and relative growth rate of height, diameter and leaf number. Forest type (wet forest and dry forests), species distribution type (dry, ubiquitous and wet species) were included in the models as fixed factors. Species, plot and their interaction were included as random factors. Model with the lowest Akaike information criteria (AIC) was selected for each dependent variable. To assess the physiological performance of species with contrasting distribution in each forest, repeated measures ANOVA was carried out separately for each forest site using the average measurements for the two dry seasons. Values of absolute growth in height, diameter, leaf number, and stomatal conductance were log_10_‐transformed to achieve normality and stability of the variance. Leaf water potential and soil matric potential were also log‐transformed as −Log (−*Ψ* + 1).

Species survival could not be evaluated using the normal Kaplan–Meier survival analysis because most of the species in both the dry and wet forests had more than 50% survival at the end of the experiment. Survival was therefore calculated as the proportion of seedlings that survived at the end of 2 years relative to survival 2 months after planting in the field. The effects of forest type and species distribution type on survival were tested with a generalized linear mixed‐effects model with forest and species distribution as fixed effects and plots and species as random effects.

Effect sizes of individual factors were calculated as the sum of square of the effect divided by the total sum of square of all effects in the model and their errors terms excluding the sum of square for the intercept. For the repeated measure ANOVAs effect sizes were calculated separately for the within‐subjects effects and between‐subjects effects, hence total effects size for the between‐subjects and within‐subjects effects will be greater than 100%. Relative drought survival performance in the field was quantified as the ratio of percent survival in the dry forest relative to wet forest (Engelbrecht & Kursar, 2003). Similarly, relative drought growth performance was quantified as the ratio of absolute growth rate in the dry forest over absolute growth rate in the wet forest. To evaluate whether species performance (survival and growth) in the field is a good indicator of species position on the rainfall gradient, a Spearman's rank correlation was conducted between relative field performance and species rainfall minimum and optimum as determined from individual species response curves (see Amissah et al., 2014). Statistical analysis was conducted using IBM SPSS software version 23.

## 3 RESULTS

### 3.1 Variation in climatic conditions and soil fertility

**Figure 1:**
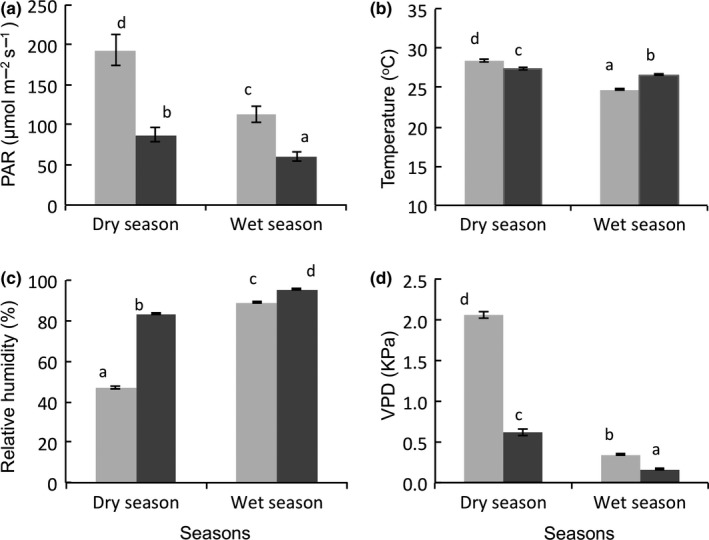
Variation in environmental variables in dry and wet seasons between dry forest (gray bars) and wet forest (black bars): (a) Photosynthetically active radiation (PAR), (b) temperature, (c) relative humidity, and (d) vapor pressure deficit. Means and standard errors are shown. Bars accompanied by different letter are significantly different at *p* ˂ 0.05 (Bonferroni post hoc test)

Environmental conditions (PAR, temperature, relative humidity, VPD) varied significantly between forest type and season (Table 1), and all variables but PAR showed a significant interaction between forest type and season (Table 1, Figure 1). PAR and VPD were higher in the dry forest than in the wet forest, and in the dry season than in the wet season, whereas the reverse was the case for RH (Table 1, Figure 1). The interaction between forest type and season indicated that, in general, the difference between the two forest types was most marked in the dry season. For both forest types, temperature was higher in the dry season than in the wet season (Figure 1b), but in the dry season, the temperature was higher in the dry forest than in the wet forest. In the wet season, the reverse was the case (Figure 1b).

**Table 1:** Seasonal variation in photosynthetically active radiation, temperature, relative humidity and vapor pressure deficit between dry and wet forests

Variable	Forest type (FT) (%)	Seasons (S) (%)	S × FT (%)
PAR	49.9*** (64.0)	18.9*** (38.9)	1.6 (3.4)
Temperature	9.4** (25.1)	260.6*** (65.5)	109.0*** (27.4)
Relative humidity	2023.4*** (98.6)	5567.8*** (76.3)	1700.1*** (23.3)
VPD	664.4*** (70.9)	1485.2*** (73.8)	497.9*** (24.7)

*Notes*

The table shows the results of ANOVA with season as repeated measure, forest type (FT) as independent variable and PAR, temperature relative humidity and vapor pressure deficit as dependent variables. *F* values for within‐ and between‐subjects effects are given. *n* = 15 for all three dependent variables for each forest type. Effect size (*η* ^2^) for each independent variable is given in parentheses.

Significance of *F* values are given as **p ≤ 0.01; ***p ≤ 0.001.

Soil from the wet forest had higher pH and lower N, C, available P and K, base saturation and exchangeable cations (Ca, Mg, K and Na) than soil from the dry forest (Supporting Information Appendix S4), which indicates that soils from the wet forest are less fertile than soils from the dry forest.

### 3.2 Variation in soil water potential

**Figure 2:**
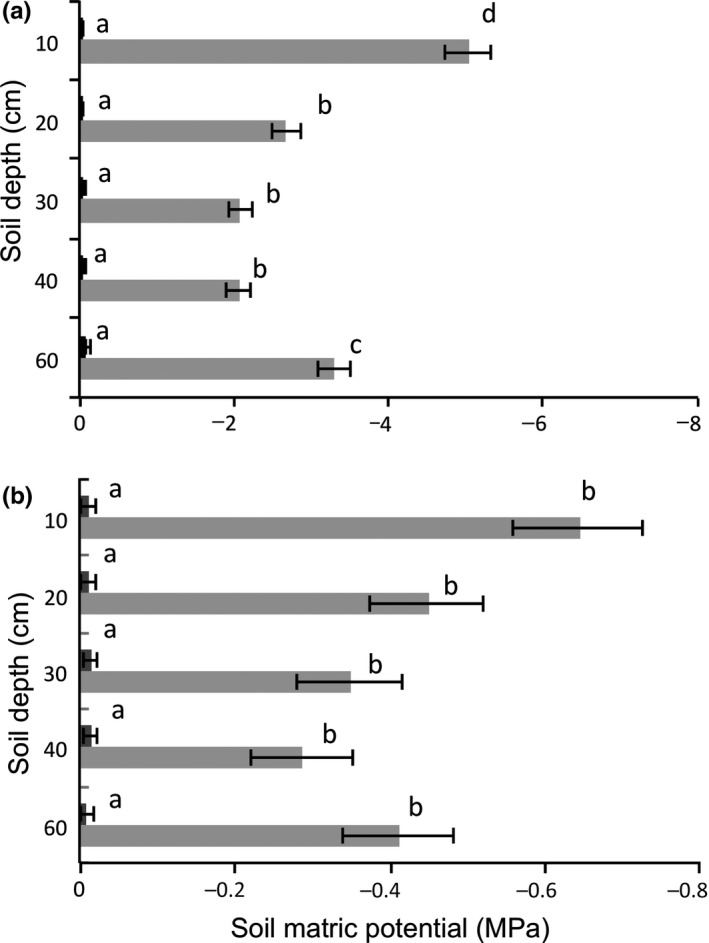
Dry season (gray bars) and wet season (black bars) variation in soil matric potential in (a) dry forest and (b) wet forest. Means and standard errors are shown. Bars accompanied by different letters are significantly different at *p* ˂ 0.05 (Bonferroni post hoc test). Please note that the scaling of the *x*‐axis differs between the two panels

Soil matric potential (*Ψ*_soil_) varied significantly with forest type (effect size of 81.3%), season (69.9%), and the interaction between forest type and season (24.1%, Table 2, Figure 2). In the wet season, both forests had similarly high soil water potential close to zero (−0.01 MPa in the wet forest, −0.04 MPa in the dry forest), indicating a high plant water availability. In the dry season, the *Ψ*_soil_ was lower, indicating water stress. The average dry season soil water potential across the soil profile was especially low in the dry forest (−3.03 MPa) compared to the wet forest (−0.43 MPa), and this difference was even more marked in the topsoil, being −5.02 MPa in the dry forest (Figure 2a) and −0.64 MPa in the wet forest (Figure 2b). In the dry season, there was for both forests a tendency for soil water availability to increase from the topsoil (10 cm depth) to 40 cm depth, and then to decrease again at 60 cm depth, but this pattern was only significant in the dry forest (Bonferroni post hoc test, *p* < 0.001).

**Table 2:** Seasonal variation in soil matric potential (*Ψ*_soil_) between dry and wet forests

Variable	Forest type (FT)	Seasons (S)	Soil depth (SD)	FT × SD	S × FT	S × SD	S × FT × SD
*Ψ*_soil_	978.1*** (81.3)	2968.4*** (69.9)	23.7*** (7.8)	7.4*** (2.4)	1022.4*** (24.1)	30.3*** (2.9)	8.2*** (0.7)

*Notes*

The table shows the results of ANOVA with season as repeated measure, forest and soil depth as independent variable and *Ψ*_soil_ as dependent variable. *F* values for within‐ and between‐subjects effects are given. *n* = 55 for each forest type. Effect size (*η* ^2^, in %) for each independent variable is given in parentheses.

Significance of *F* values are given as ***p ≤ 0.001.

### 3.3 Variation in leaf physiology and relation with growth rate

The three physiological variables (stomatal conductance, predawn and mid‐day leaf water potential) varied significantly with forest type and season (average effect size is 32%), the interaction between forest type and season and to a lesser extent with species distribution (Table 3). There was no significant interaction between forest type and species distribution (Table 3), indicating that the three distribution groups showed a similar ranking in physiological performance in both forest types. However, there was significant interaction when we used species as a factor in the analysis (Supporting Information Appendix S5). Additionally, stomatal conductance was more influenced by species distribution than by season and there was an interaction between season and species distribution (Table 3).

**Table 3:** Seasonal variation in predawn (*Ψ*_pd_), mid‐day leaf water potential (*Ψ*_mid_) and stomatal conductance between dry and wet forests

Variable	Forest type (FT)	Seasons(S)	Species distribution (SPD)	FT × SPD	S × FT	S × SPD	S × FT × SPD
Leaf water potential_pd_	270.5*** (33.3)	731.9*** (45.4)	25.*** (6.1)	1.2 (0.3)	383.6*** (23.8)	2.4 (0.3)	1.5 (0.2)
Leaf water potential_md_	73.8*** (11.)	893.8*** (49.8)	38.2*** (11.9)	0.8 (0.3)	406.3*** (22.7)	2.4 (0.3)	0.7 (1)
Stomatal conductance	274.2*** (50)	3.0 (0.6)	14.7*** (5.3)	0.5 (0.2)	257.8*** (48.7)	4.7** (1.7)	5.3** (2.0)

*Notes*

The table shows the results of ANOVA with season as repeated measure, forest type, and species distribution (dry, ubiquitous, and moist/wet) as independent variable and *Ψ*_pd_ and *Ψ*_mid_ as dependent variables. *F* values for within‐ and between‐subjects effects are given. Effect size (*η* ^2^, in %) for each independent variable is given in parentheses.

Significance of *F* values is given as **p ≤ 0.01; ***p ≤ 0.001.

**Figure 3:**
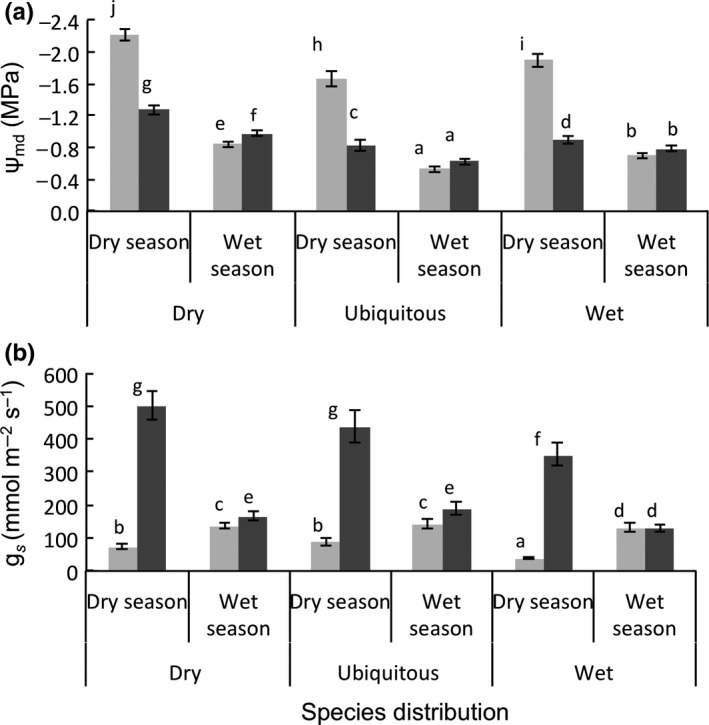
Seasonal variation in leaf physiology among species with different distribution types (dry forest species, ubiquitous species, and wet forest species) in dry (gray bars) and wet (black bars) tropical forests. (a) Mid‐day leaf water potential (*Ψ*_mid_) and (b) stomatal conductance (*g*_s_). Means and standard errors are shown. Bars accompanied by different letters are significantly different at *p* ˂ 0.05 (Bonferroni post hoc test)

Predawn leaf water potential indicates how plants are in equilibrium with the soil. Predawn leaf water potential was significantly lower in the dry forest than in the wet forest and in the dry season than in the wet season, which shows that these plants indeed experience low plant water availability (Supporting Information Appendix S6). The mid‐day leaf water potential indicates the maximum amount of soil and atmospheric drought stress that plants experience. Mid‐day leaf water potential (Figure 3a) followed a similar pattern as predawn leaf water potential. In the dry forest, all distribution groups had more negative mid‐day leaf water potential and a lower stomatal conductance in the dry season compared to the wet season, and this seasonal decrease in stomatal conductance was even stronger for the wet forest species (Figure 3b). In both forest types and seasons, dry forest species had a significantly more negative *Ψ*_mid_ than the ubiquitous and wet species (Figure 3a), which indicates that dry species are able to tolerate more drought stress. Stomatal conductance (*g*_s_) was significantly higher in wet than in dry forest, and there was a significant interaction between forest and season (Table 3). In the dry forest, stomatal conductance decreased in the dry season (Figure 3b) whereas in the wet forest stomatal conductance increased in the dry season (Figure 3b). This is consistent for all species groups and is consistent with the significant interaction between forest types, seasons and species groups for stomatal conductance shown in Table 3. In general, dry and ubiquitous species had significantly higher stomatal conductance than the wet species (Figure 3b). Dry season stomatal conductance in the wet forest correlated positively with absolute growth rate in height (Pearson *r *=* *0.55, *N* = 23, *p *=* *0.006) and with relative growth rate in height (Pearson *r *=* *0.53, *N* = 23, *p *=* *0.009). In contrast, dry season stomatal conductance in the dry forest did not correlate with absolute growth rate in height (Pearson *r *=* *0.13, *N* = 23, *p *=* *0.551 and relative growth rate in height (Pearson *r *=* *0.34, *N* = 23, *p *=* *0.111).

### 3.4 Seedling growth and survival in dry and wet forests

**Figure 4:**
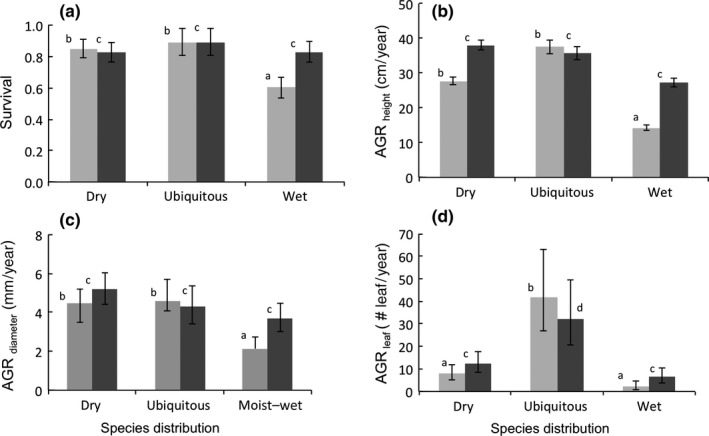
Absolute growth rate and survival of species with different distributions (dry forest species, ubiquitous species, and wet forest species) in dry (gray bars) and wet (black bars) tropical forests at the end of 2‐year period. (a) Survival, (b) height growth, (c) diameter growth, and (d) number of leaves. Mean and standard errors are shown. Bars accompanied by different letters are significantly different at *p* ˂ 0.05 (least significant difference post hoc test). The different letters represent significance within each forest site

Seedling survival at the end of 24 months was significantly higher in wet compared to dry forest, and there was a significant species distribution x forest type interaction (Table 4, Figure 4a).

**Table 4:** Detailed results of linear mixed models (growth) and generalized linear mixed models (survival) of the effects of forest site, species distribution on six growth parameters and survival at 24 months

Variable	Forest	Species distribution	Forest × species distribution	Species	Plots	Species × plots
AGR_height_	8.47**	3.45*	24.03***	3.09**	3.25**	7.80***
AGR_diameter_	4.2*	2.34	26.34***	3.11**	3.40**	8.39***
AGR_leaf number_	0.01	7.36**	1.83	3.12**	2.99**	8.63***
RGR_height_	16.70***	1.56	17.71***	3.10**	3.40**	7.308***
RGR_diameter_	1.77	1.81	25.28***	3.12**	3.39**	6.75***
RGR_leaf_ _number_	7.23*	7.69**	18.95**	3.08**	3.13**	8.29***
Survival_24mths_	17.01***	1.6	27.0***	2.70**	0.85	NA

*Notes*

AGR: absolute growth rate; NA: not determined; RGR: relative growth rate.

Plots and species were used as random variables. The tables present the *F* values and *Z* values for the fixed and random effects and their significant levels.

***p ≤ 0.001, **p ≤ 0.01, *p ≤ 0.05

Seedling growth varied significantly with forest type (for absolute growth in height and diameter), species distribution type (for absolute growth in height and leaf number), and their interaction (for absolute growth in height and diameter) (Table 4). AGR_height_ and AGR_diameter_ were significantly higher in wet compared to dry forest (Figure 4b,c), whereas absolute growth rate in leaf number did not vary with forest type (Table 4). Comparison of species growth within dry and wet forest sites showed contrasting results. In the dry forests, there were significant differences among species distribution types (Table 5). Dry forest species showed a higher absolute height and diameter growth rate than wet forest species but showed similar growth to ubiquitous species. In contrast, species distribution types growing in wet forest did not show significant differences in absolute height and diameter growth (Table 4, Figure 4b,c). In both forests absolute growth in leaf number showed significant difference; ubiquitous species tended to grow leaves faster, whereas dry and wet species realized similar growth (Table 4, Figure 4d). Except for leaf number, patterns in relative growth rate were similar to those of absolute growth rate (Table 4, Supporting Information Appendix S7).

**Table 5:** Comparison (least significance difference) of the performance of species distribution types within dry and wet forest sites

Variable	Within dry forest Species distribution	Within wet forest Species distribution
AGR_height_	0.01	ns
AGR_diameter_	0.02	ns
AGR_leaf number_	ns	ns
RGR_height_	0.04	ns
RGR_diameter_	0.02	ns
RGR_leaf_ _number_	ns	0.07
Survival_24mths_	0.02	ns

*Notes*

ns, not significant.

Comparison is presented for only dry and wet species growing in the dry and wet forest sites. Values shown are significant levels.

### 3.5 Species performance versus species rainfall distribution

**Figure 5:**
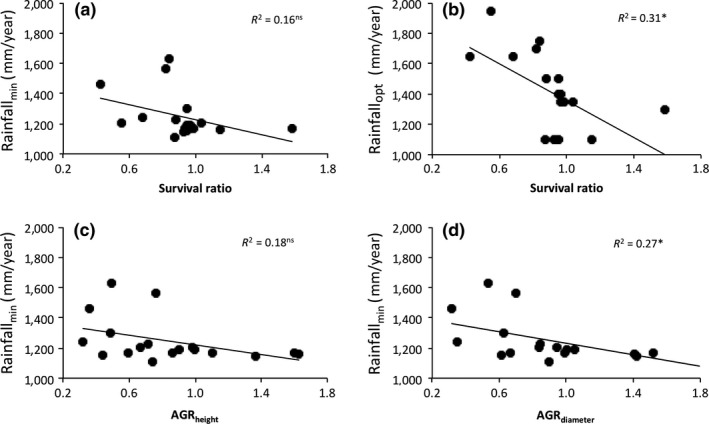
Correlation between species position along rainfall gradient and species drought performance and growth rates in the field. Drought performance in the field was quantified in terms of survival as the ratio of percent survival in the dry forest relative to percentage survival in the wet forest. Survival for each forest was calculated as the number of seedlings surviving at the end of 2 years relative to number of seedlings alive 2 months after planting in the field (in %). Growth performance in the field was quantified as the ratio of absolute growth rates in dry forest relative to the absolute growth rates in the wet forest. (a) rainfall minimum and survival ratio, (b) rainfall optimum and survival ratio, (c) rainfall minimum and ratio of absolute growth rate of height (AGR_height_), and (d) rainfall minimum and ratio of absolute growth rate of diameter (AGR_diameter_). Regression lines, coefficients of determination (*R* ^2^), and significance level (*p < 0.05) and nonsignificant (ns) are shown. The correlation is based on 18 species whose response curves were constructed (Amissah et al., 2014). Inventory data were not available for the other five species; hence, their response curves could not be constructed

Relative drought survival (i.e., the ratio of survival in the dry forest over survival in the wet forest) in the field was significantly negatively correlated with optimum annual rainfall at which the species occur (Spearman *r *=* *−0.59, *p *=* *0.010, *N *=* *18, Figure 5b) and tended to correlate negatively with the minimum annual rainfall at which species occur (Spearman *r *=* *−0.47, *p *=* *0.051, *N *=* *18, Figure 5a). Similar relationships were found between species relative growth performance under dry conditions and the minimum rainfall at which they occurred. The ratio of absolute diameter growth rates in dry forest over wet forest showed significant negative relationship with a species rainfall minimum (Spearman *r *=* *−0.56, *p *=* *0.015, *N *=* *18, Figure 5d), and the ratio of absolute height growth rate in dry forest over wet forest also tended to show negative relationship with a species rainfall minimum (Spearman *r *=* *−0.47, *p *=* *0.050, *N *=* *18, Figure 5c).

## 4 DISCUSSION

We investigated how seedling physiology, growth and survival varied between dry and wet forest sites, between dry and wet seasons, and between different rainfall distribution groups. Species relative performance in the field was related to species distribution along the rainfall gradient. Drought led to reduced stomatal conductance in the dry season, especially in the dry forest but in the wet forest stomatal conductance increased in the dry season. This led to reduced growth and survival in all species, more strongly so for the wet forest species in the dry forest. Relative drought performance could predict species position on rainfall gradients, in which species that grow and survive relatively well in dry environments have their optimum distribution in drier areas.

### 4.1 Environmental conditions: the dry forest experiences higher irradiance and fertility, but also stronger seasonality and drought stress

We hypothesized and found that the dry forest experiences more soil and atmospheric drought stress than the wet forest and that this difference is more pronounced in the dry season. As expected, PAR and VPD were higher in the dry forest than in the wet forest and also higher in the dry season than in the wet season (Figure 1), because under dry conditions the forest canopy is (seasonally) more open, especially in dry forests such as in our study site where there are more deciduous species and less cloudiness (Hall & Swaine, 1981; Swaine & Becker, 1999; Wright & Van Schaik, 1994). In our study, the atmospheric drought stress caused by VPD was higher in the dry forests than in the wet forest, both in the dry season (2.0 vs. 0.62 kPa) and in the wet season (0.35 vs. 0.16 kPa). This result is consistent with results from wetter Amazonian rain forest sites where larger VPD was observed in the dry season than the wet season (Costa et al., 2010).

An increased VPD leads to increased evapotranspiration and in combination with less rain result in the dry season soil matric potential, which was on average more negative in the dry forest (−3.03 MPa, Figure 2a) than in the wet forest (−0.43 MPa, Figure 2b). The results obtained here are consistent with our first hypothesis. Other studies also found more negative soil matric potentials in the dry season (Markesteijn, Iraipi, Bongers, & Poorter, 2010; Veenendaal, Swaine, Agyeman et al., 1996; Wright & Cornejo, 1990).

Soil from the wet forest had lower N, P and exchangeable cations than the dry forest (Supporting Information Appendix S4) indicating lower fertility of the wet forest soils. Other studies in Ghana (Veenendaal Swaine, Lecha et al., 1996) and to a lesser extent in Panama (Condit, Engelbrecht, Pino, Pérez, & Turner, 2013) also found lower nutrients levels in wet evergreen forest than the moist semideciduous forest, probably because of deeper weathering of the bedrock material, and stronger nutrient leaching (Swaine, 1996).

### 4.2 Leaf physiology: drought leads to reduced physiological activity, but dry forest species have higher gas exchange and physiological drought tolerance than wet species

We hypothesized that because of stronger drought stress in the dry forest, seedlings growing in the dry forest would have lower leaf water potential and stomatal conductance than seedlings growing in the wet forest, especially in the dry season. Increased edaphic and atmospheric drought during the dry season indeed resulted in increased plant water stress, as reflected in a lower predawn and mid‐day leaf water potential (Supporting Information Appendix S6, Figure 3a), which, in turn, led to reduced stomatal conductance (Figure 3b) thus reducing water loss. This especially occurred for wet forest species in dry forest site. Stomatal conductance, however, increased in the dry season in the wet forest contrary to our prediction. In the wet forest, the dry season was rather short and less intense (Supporting Information Appendix S3) and higher irradiance during the less cloudy dry season and modestly higher VPD (0.6 kPa) may have contributed to a higher stomatal conductance in the dry season. Reduced stomatal conductance obtained for all species in our dry forest supports other studies, which recorded for all species reduced stomatal conductance during the dry season (Choat, Ball, Luly, Donnelly, & Holtum, 2006; Craven et al., 2011). In the dry season, the soils in the upper profile are too dry to compensate for increased water demand resulting in more negative water balance in plants, especially for plants possessing shallow roots and species with an anisohydric strategy (Parolin, Lucas, Piedade, & Wittmann, 2010). The higher VPD and temperature recorded in our study in the dry season and in the dry forest might play a large role in determining stomatal conductance, potential assimilation and therefore the carbon balance of plants (Duff et al., 1997; Jones, 1992; Myers et al., 1997). Certain plant functional types have been found to show acclimation to long‐term VPD and therefore do not experience reduced stomatal conductance in response to short‐term VPD. In such instances, plants may maintain carbon gain despite increases in VPD (Marchin, Broadhead, Bostic, Dunn, & Hoffmann, 2016). Increased VPD in our dry forest that resulted in reduced stomatal conductance may contribute to reduced growth in the dry forest.

The ability of plants to reduce transpiration and to prevent xylem cavitation by having a higher embolism resistance is critical to plant survival and growth in dry environments (Brodribb & Cochard, 2009; Choat et al., 2012; Markesteijn, Poorter, Paz, Sack, & Bongers, 2011; Sterck, Markesteijn, Toledo, Schieving, & Poorter, 2014). Drought markedly decreases physiological activity of seedlings both in controlled studies and in the field and hence has a negative impact on seedling growth and survival (Amissah, Mohren, Kyereh, & Poorter, 2015; Craven et al., 2011; Parolin et al., 2010). In our study dry season stomatal conductance correlated positively with relative and absolute growth rates, which supports results of a study carried out in an Indian tropical dry forest in which stomatal conductance was found to explain 62% of variation in relative growth rate; indicating that stomatal conductance plays an important role in shaping growth patterns across spatial and temporal gradients of soil water availability (Chaturvedi, Raghubanshi, & Singh, 2012).

None of the three physiological variables evaluated showed a significant interaction between forest and species distribution. Thus there was no home advantage of species in their physiological performance in contrast with our predictions. In both forest types and seasons, dry and ubiquitous species had significantly higher stomatal conductance than the wet species (Figure 3b), indicating that they are physiologically more active and can realize potentially a high carbon gain as demonstrated by the result of this study. At the same time, dry forest species had a significantly more negative leaf water potential than the ubiquitous and wet species, while exhibiting a slightly higher stomatal conductance. This suggests that dry forest species have either superficial roots and experience more drought stress, or that they follow an anisohydric strategy and can tolerate more drought stress. Anisohydric species keep their stomata open for a longer period and to realize a higher carbon gain, but this comes at the expense of a lower water potential during the dry season (Sade et al., 2012). Dry forest species seem therefore to combine the best of both worlds; they are not only physiologically more active, but they are also physiologically more drought tolerant.

### 4.3 Growth and survival: dry forest species are super performers and have a home advantage

We hypothesized that species would have a distribution‐based home advantage, with dry species performing better in the dry forest, and wet species performing better in the wet forest. In general, survival and growth were higher in the wet forest compared to the dry forest (Figure 4a–d), despite the lower soil fertility and irradiance. This suggests that across these forests, plant water availability and the length of the rainy period are the main drivers affecting plant performance in forest gaps. Similarly, growth of Asian trees, occurring in seasonal forest, was significantly positively correlated with dry season precipitation levels (Vlam, Baker, Bunyavejchewin, & Zuidema, 2014).

Dry forest species indeed had a home advantage in the dry forest and had higher growth and survival rate than wet forest species (Figure 4) which is in agreement with our third hypothesis. This may reflect the inherent growth strategy of the species, as most dry species are fast‐growing light‐demanding species; of the 10 dry forest species included in this study, 20% is pioneer, 70% is nonpioneer light demander (seeds germinate in shade but seedlings need light to establish), and only 10% is shade‐tolerant. It can be reasoned that a nonpioneer light demander of the dry forest is inherently more light demanding than a nonpioneer light demander of the wet forest (and the same applies to pioneers). Light‐demanding species might especially be successful in dry forests, as irradiance levels in dry forest gaps are twice as high compared to wet forest gaps (Figure 1). These light‐demanding dry forest species combine fast gas exchange and growth with the ability to tolerate low leaf water potentials in the seedling stage. Many of these light‐demanding species are evergreen in the seedling stage but become drought deciduous and hence, drought avoiding in the adult stage (L. Poorter, personal observation). As much as 60% of the dry forest species is (brevi) deciduous as an adult. Such a deciduous, drought avoiding strategy is very costly, as with leaf abscission about half of the leaf nutrients and most of the carbon are lost (Zhang, Zhang, Chen, Zhang, & Poorter, 2015). Deciduous species are therefore mostly confined to habitats where light and nutrient availability are high (Givnish, 2002), such as in our dry forest, thus permitting the replacement of lost leaves.

We hypothesized that ubiquitous species should have an intermediate performance between dry and wet species, but they survived equally well, although they were not able to tolerate low leaf water potentials as much as the dry forest species. Three reasons can explain the success of these ubiquitous species. First, most of them are similarly light demanding as the dry forest species. Second, ubiquitous species perform equally well as dry forest species, because they have to be able to go through the same dry season bottleneck as the dry forest species. Third, it could be that these ubiquitous species reflect the widespread, “ruderal” generalist species that have recolonized West Africa successfully after the last ice ages (Holmgren & Poorter, 2007).

Our results partially contrast with those of Baltzer and Davies (2012) who did a transplanting experiment in dry (i.e., seasonal forest with 2–3 months per year <100 mm rainfall per month, total rainfall 2,700 mm/year) and wet (i.e., aseasonal forest with no month with less than 100 mm rainfall per month, total rainfall 1,950 mm/year) forest in Malaysia. In their study, height and biomass growth rates did not vary between both forest types. Additionally, widespread and aseasonal species performed similarly in the seasonally dry forest. However, in the wet forest, wet species had significantly greater relative height growth rates compared with widespread dry species (Baltzer & Davies, 2012) indicating that wet species had a home advantage. Hence, their study carried out at the wetter part of the rainfall gradient also shows a home advantage for one species group (the wet species), whereas our study carried out at the drier part of the rainfall gradient also shows a home advantage for one species group (the dry species) but not for both. In our study, dry and ubiquitous species had higher growth in dry forest than wet species. In addition, all three species distribution type had similar growth in the wet forest. This is in contrast with findings from a greenhouse experiment of Brenes‐Arguedas et al. (2008) in Panama, in which dry‐distribution species (<2,000 mm of rainfall per year) grew on average slower than wet‐distribution species (which occur in areas up to 3,000 mm of rainfall per year). They attributed the slower growth rates to a cost of drought adaptations. Furthermore, it was hypothesized that inherently slow growth rate of some dry‐distribution tropical species is one of the main factors limiting their colonization of wetter sites along the rainfall gradient (Brenes‐Arguedas et al., 2008). Our results do not support this hypothesis, but it should be mentioned that dry and wet are clearly relative terms, as our Ghanaian forests occur at the lower end of the rainfall gradient, and our wet forest is definitely less seasonal, but in terms of annual rainfall only somewhat wetter than the Panamanian dry forest. Additionally, our study comprised mostly gap species with a high representation of nonpioneer light demanders whereas their study comprised mostly shade‐tolerant species. Shade and gap species have completely different strategies, which could have influenced the results of our study. It is also possible that distribution‐based home advantage would have become clearer if the experiment had been carried out in the forest understory, or when species advance in age. Probably at a later age and larger tree sizes, some species may be outcompeted in areas that are not their home range (example, wet forest species may be outcompeted by dry forest species in dry forest and vice versa). In a reciprocal transplanting experiment with a ubiquitous temperate herbaceous species in Sweden and Italy, there was a strong advantage to local population at the two sites used providing a strong evidence of adaptive differentiation of the natural population of the species used (Ågren & Schemske, 2012).

The results obtained here have implication for climate change adaptation of the forests in Ghana. The fact that the rainfall gradient in Ghana is not as strong as rainfall gradients in other studied regions and the dry species studied here are not constrained in terms of growth in “wet” areas may explain why a recent study (Fauset et al., 2012) found a shift in species composition in these forests (a replacement of shade‐tolerant species with large deciduous nonpioneer light‐demanding canopy species in the forest reserves studied).

### 4.4 What excludes dry species from the wet forest?

The question that remains is what excludes dry species from the wet forest? In the introduction, we stated that rainfall gradients are complex gradient (in particular the rainfall gradient of Ghana), along which not only water availability, but also nutrient availability, pest pressure, and irradiance vary.

Our results show that at the seedling stage the low soil fertility in the wet forest does not limit the growth of dry species in the wet forest (cf. Baker, Burslem, & Swaine, 2003). Similarly, in controlled greenhouse experiments in Ghana and Panama only few species showed a significant growth response to soil fertility and a home–soil‐based advantage (Brenes‐Arguedas et al., 2008; Veenendaal & Swaine, 1998; Veenendaal Swaine, Lecha et al., 1996; L. Amissah et al., unpublished data). Furthermore, field transplant experiments of other studies did not provide evidence for a home–soil advantage to species with contrasting distribution (Brenes‐Arguedas et al., 2009; Gaviria et al., 2017; Swaine et al., 1997). Perhaps nutrient limitation may hamper growth when plants become older and require more nutrients for rapid growth (cf Baker et al., 2003) and also when plants become deciduous in the adult stage.

A supposedly higher herbivore pressure in wet forest neither seems to exclude dry forest species in Ghana. In our experiment, we evaluated for a 2‐month period herbivory rates and found, in contrast to the herbivore pressure hypothesis, that herbivory rates were slightly higher in the dry than in the wet forest (S. Sportel and L. Amissah et al., unpublished data). In the dry forest, dry forest species suffered from higher herbivory rates than ubiquitous and wet species (S. Sportel and L. Amissah et al., unpublished data), which suggest that they actually have a home *dis*advantage and that pest pressure cannot explain the distribution of tree species. On the contrary Spear, Coley, and Kursar (2015) found a greater risk of pathogen‐caused damage and mortality in the wetter forests than in drier forests. Their study also showed dry forest species tended to suffer more pathogen‐caused mortality than wet forest species in both the dry and wet forests studied. Consequently, pathogens damage could in part act as a biotic filter limiting the recruitment of some dry forest species in the wetter forests (Spear et al., 2015).

It is also possible that a distribution‐based home advantage could become clearer with experiments carried out in the forest understory, rather than in gaps and also with a high proportion of shade‐tolerant species. In the wet forest, fast‐growing light‐demanding dry forest species may realize faster growth rates than wet species in the high light environment of gaps, but they would probably not be able to cope with the deeper shade in the understory.

### 4.5 Species drought performance predicts species position along the rainfall gradient

Species drought performance (growth and survival) in the dry forest relative to the wet forest was a significant predictor of the minimum and optimum rainfall at which species occurred (Figure 5b,d). This result was consistent with our hypothesis. Hence, species that performed relatively well in dry forest sites or under dry conditions tend to occur in drier forests (cf. Baltzer et al., 2008; Engelbrecht et al., 2007; Poorter & Markesteijn, 2008; Sterck et al., 2014). These findings suggest that seasonal drought plays an important role in a species’ distribution. However, other factors such as dispersal limitation and disturbance may play a role in species distribution as a large part of the variation remains unexplained. A modeling study on trees in Spain has shown that growth and mortality are indeed major determinants of species distributions (Garzon, Benito, & Zavala, 2013). However, a modeling study by Sterck et al. (2014) found that species distribution along a rainfall gradient was not related to the growth potential of the species, but to species survival under suboptimal conditions (i.e., their water compensation point).

## 5 CONCLUSIONS

In the dry forest and the dry season, drought leads to reduced gas exchange, growth and survival of plants. Dry forest species outperform wet species at their habitat of origin. Wet forest species perform (growth and survival) comparably with dry and ubiquitous species at their habitat of origin but reduce stomatal conductance more strongly than dry species under drought especially in dry forests. Drought performance in the dry forest relative to the wet forest significantly predicted species position on the rainfall gradient, which indicates that seasonal drought acts as an environmental filter in shaping species distribution. Consequently, alteration of seasonal patterns of rainfall in many tropical forests as a result of climate change may affect regeneration of species, cause a shift in tree productivity, species composition, and distribution.

## CONFLICT OF INTEREST

None declared.

## AUTHOR CONTRIBUTIONS

LA, GMJM, LP, BK, and VKA conceived the idea. LA collected the data, performed the analysis, and wrote the first draft of the manuscript. All authors contributed substantially to revisions.

## DATA ACCESSIBILITY STATEMENT

The authors intend to make their data accessible and would deposit their data in Dryad. We would like to place restrictions on accessibility of the data for a period of up to a year after publication.
