# Supplementary material for: Rainfall seasonality and drought performance shape the distribution of tropical tree species in Ghana
Source: Ecol Evol. 2018 Jul 30;8(16):8582–97. doi: 10.1002/ece3.4384 (PMC6144999; doi:10.1002/ece3.4384)
Supplement: Supplementary file 1 [file ECE3-8-8582-s001.docx]

Appendix S1. Map showing the distribution of plots in the two forests used for the reciprocal planting of 23 species and their location in Ghana. (a) dry forest and (b) wet forest.

Appendix S2. List of species, abbreviations, family, guild and forest type. Species’ family name, guild and forest types are according to (Hall and Swaine 1981, Hawthorne 1995, Hawthorne & Jongkind 2006a, Hawthorne and Ntim Gyakari 2006b). NPLD = Non-pioneer light demander. *= species whose response curves are not available.

| Species | Family | Species guild | Forest type |
| --- | --- | --- | --- |
| *Heritiera utilis* Sprague | Sterculiaceae | NPLD | Wet |
| **Lophira alata* Banks ex Gaertn. | Ochnaceae | Pioneer | Wet |
| **Khaya ivorensis A. Chev.* | Meliaceae | NPLD | Wet |
| *Pentadesma butyracea* Sabine | Caesalpinaceae | Shade tolerant | Wet |
| *Entandrophragma angolense* (Welw). DC | Meliaceae | NPLD | Moist |
| *Turraeanthus africanus* (Welw. ex C.DC.) Peller | Meliaceae | Shade tolerant | Moist |
| **Tieghemella heckelii* Pierre ex Chev | Sapotaceae | NPLD | Moist |
| *Terminalia ivorensis_Wet_* A.Chev. | Combretaceae | Pioneer | Ubiquitous |
| *Piptadeniastrum africanu*m (Hook.f.) Brenan | Mimosaceae | NPLD | Moist |
| *Ricinodendron heudeloti*i (Baill.) Pierre ex Pax | Euphorbiaceae | Pioneer | Dry |
| *Newbouldia laevis* (P.Beauv.) Seeman ex Bureau | Bignoniaceae | Pioneer | Dry |

Appendix S2 continued

| Species | Family | Species guild | Forest type |
| --- | --- | --- | --- |
| *Mansonia altissima* (A.Chev.) A.Chev. | Sterculiaceae | NPLD | Dry |
| *Nesogordonia papaverifera* (Hook.f.) Brenan | Sterculiaceae | Shade tolerant | Dry |
| *Ceiba pentandra* (Linn.) Gaertn. | Bombacaceae | NPLD | Dry |
| **Celtis zenkeri Engl.* | Ulmaceae | NPLD | Dry |
| *Albizia zygia* (DC.) J.F. Macbr. | Mimosaceae | NPLD | Dry |
| *Pericopsis elata* (Harms) Van Meeuwen | Papilionaceae | NPLD | Dry |
| *Sterculia rhinopetala* K. Schum | Sterculiaceae | NPLD | Dry |
| **Khaya anthotheca* (Welw.) C. DC. | Meliaceae | NPLD | Dry |
| *Antiaris toxicaria* Leschenault | Moraceae | NPLD | Ubiquitous |
| *Strombosia pustule* J.Leonard | Olacaceae | Shade tolerant | Ubiquitous |
| *Terminalia ivorensis* A.Chev. | Combretaceae | Pioneer | Ubiquitous |
| *Terminalia superba* Engl. and Diels | Combretaceae | Pioneer | Ubiquitous |

AppendixS3.Seasonal variation in monthly rainfall from July 2011-June 2013 in (a) dry forest and (b) wet forest. Monthly rainfall was compiled from daily rainfall collected from a manual rainfall gauge placed about 3 km from each forest.

Appendix S4.Acidity and nutrient concentrations and mechanical properties of soils of two contrasting forest sites (dry forest and wet forests) in Ghana.

| Soil nutrients and texture | Dry forest | Wet forest |
| --- | --- | --- |
| pH (H_2_O 1:1) | 6.20 | 4.88 |
| Carbon (%) | 1.97 | 1.75 |
| Nitrogen (%) | 0.18 | 0.14 |
| Organic matter (%) | 3.40 | 3.02 |
| Exchangeable Cation  Ca ( Cmol/kg) | 8.54 | 1.60 |
| Mg ( Cmol/kg) | 3.20 | 0.53 |
| K ( Cmol/kg) | 0.15 | 0.08 |
| Na ( Cmol/kg) | 0.05 | 0.03 |
| TEB Cmol/Kg | 11.94 | 2.24 |
| ECEC (Cmol/Kg) | 12.04 | 3.09 |
| Base Saturation (Cmol/Kg) | 99.17 | 72.49 |
| Available  P (ppm) | 4.94 | 3.75 |
| K (ppm) | 62.90 | 34.15 |
| Mechanical properties  Clay (%) | 22.40 | 20.20 |
| Sand (%) | 23.74 | 15.56 |
| Silt (%) | 53.86 | 64.24 |
| Gravel content | - | 42.22% |
| Texture type | Silty loam | Silty loam |

**Appendix S5**.Seasonal variation in pre-dawn (Ψ_pd_), mid-day leaf water potential (Ψ_mid_) and stomatal conductance between dry and wet forests. The table shows the results of ANOVA with season as repeated measure, forest type and species as independent variables, Ψ_pd_,Ψ_mid_and stomatal conductance as dependent variables. F values for within and between subjects effects are given. Significance of F values are given as **P ≤ 0.01; ***P ≤ 0.001.

| Variable | Forest type (FT) | Seasons(S) | Species (SPP) | FT x SPP | S x FT | S x SPP | S x FT x SPP |
| --- | --- | --- | --- | --- | --- | --- | --- |
| Leaf water potential_pd_ | 756.5*** | 1236*** | 34.3*** | 6.53*** | 665.2*** | 9.9*** | 5.2*** |
| Leaf water potential_md_ | 289.8*** | 1505.1*** | 71.2*** | 2.2** | 682.8*** | 11.2*** | 4.1*** |
| Stomatal conductance | 492.1*** | 2.69 | 9.1*** | 368.0*** | 3.8*** | 4.4*** | 2.8*** |

Appendix S6.Seasonal variation in predawn leaf water potential (Ψ_pd_) among species with different distribution types (dry species, ubiquitous species and wet species) in dry (grey bars) and wet (black bars) tropical forests. Means and standard errors are shown. Bars accompanied by different letters are significantly different at P˂ 0.05 (Bonferroni Post-Hoc test).

Appendix S7. Relative growth rates of species with different distributions (dry species, ubiquitous species and moist-wet species) in dry (grey bars) and wet (black bars) tropical forests at the end of the 2-year period. (a) Height, (b) diameter and (c) number of leaves. Means and standard errors are shown. Bars accompanied by different letters are significantly different at P˂ 0.05 (Least Significant Difference Post-Hoc test).The different letters represent significance within each forest site.

Appendix S8.Pearson correlations among absolute growth in height, diameter and leaf number and relative growth rate in height, diameter and leaf number and their p values. ***P ≤ 0.001, N = 23. AGR = Absolute growth rate and RGR = relative growth rate.

|  | AGR_height_ | AGR_diameter_ | AGR_leafnumber_ | RGR_height_ | RGR_diameter_ |
| --- | --- | --- | --- | --- | --- |
| AGR_height_ |  |  |  |  |  |
| AGR_diameter_ | 0.8*** |  |  |  |  |
| AGR_leafnumber_ | 0.6*** | 0.6*** |  |  |  |
| RGR_height_ | 0.8*** | 0.6*** | 0.4*** |  |  |
| RGR_diameter_ | 0.6*** | 0.8*** | 0.4*** | 0.8*** |  |
| RGR_leafnumber_ | 0.6*** | 0.6*** | 0.9*** | 0.5*** | 0.5*** |
